# Prevalence and Impact of Central Sensitisation and Kinesiophobia on Functional Capacity and Quality of Life in Behçet’s Disease: A Cross-Sectional Study

**DOI:** 10.31138/mjr.061124.oal

**Published:** 2025-06-07

**Authors:** Gülay Alp, Fulden Sari

**Affiliations:** 1 Department of Internal Medicine, Division of Rheumatology, Uşak University School of Medicine, Uşak, Turkey; 2 Department of Physiotherapy and Rehabilitation, Faculty of Health Sciences, Bingol University, Bingol, Turkey

**Keywords:** Behçet’s disease, central sensitisation, functional capacity, quality of life, kinesiophobia

## Abstract

**Objective:**

Behçet’s disease (BD) may experience heightened pain sensitivity, potentially related to central sensitisation (CS). The hypersensitive central nervous system causes physical inactivity and kinesiophobia due to overreacting to stimuli that would not usually cause pain. This study aims to determine the frequency of CS in patients with BD and to evaluate the relationship between CS and kinesiophobia, exercise capacity, disease activity, and quality of life (QoL).

**Methods:**

The study, which included 55 patients with BD and 55 healthy controls (HCs), employed a comprehensive approach. All participants were administered the Tampa Kinesiophobia Scale (TKS), the 6-minute walk test (6MWT), and the central sensitisation inventory.

**Results:**

CS was detected in 61.4% of with BD. Among the 55 patients, 24 (45.5%) were male, with a median age of 42 years (IQR 17) and a median disease duration of 8 years (IQR 10). Compared to age- and gender-matched HCs, patients with BD exhibited higher CS, increased kinesiophobia, and shorter walking distances. There were moderate correlations between CS scores and the 6MWT, TKS, BDCAF, and BDQoL in patients with BD (Rho = 0.51, Rho = 0.56, Rho = 0.48, and Rho = 0.56, respectively; all p-values < .001). Hierarchical linear regression analysis demonstrated a significant association between QoL and the presence of CS (95% Confidence Interval (CI) 0.033–0.562, p=0.028) and kinesiophobia (95% CI 0.245–0.766, p < 0.001).

**Conclusion:**

The prevalence of CS and kinesiophobia in BD patients is a significant finding, shedding light on the factors contributing to reduced QoL and functional exercise capacity.

## Key Points

1. Central sensitisation is prevalent in Behçet’s disease (BD) and significantly impacts patients’ functional capacity and quality of life.
2. Kinesiophobia is strongly associated with reduced exercise capacity and poorer quality of life in patients with BD.
3. Addressing central sensitisation and kinesiophobia could improve both physical and psychological outcomes in BD management

## INTRODUCTION

Behçet’s disease (BD) is a rare, chronic inflammatory disorder with unique multisystem involvement, notably presenting with recurrent oral and genital ulcers and eye inflammation. This distinctiveness sets BD apart from some other rheumatic diseases, such as rheumatoid arthritis (RA) and spondyloarthritis, which typically do not have direct involvement of the central nervous system (CNS). However, not all individuals with Behçet’s disease will experience CNS involvement. CNS involvement in BD can manifest as neurological symptoms, including headaches, meningoencephalitis, cranial nerve palsies, and other neurological deficits. ^1^

Central sensitisation (CS) is when the CNS becomes more responsive to stimuli, leading to heightened sensitivity and increased pain perception. Pain in BD can be diverse, ranging from oral and genital ulcers to neurological symptoms associated with CNS involvement. Unfortunately, the specific mechanisms triggering and perpetuating pain in BD are not yet fully elucidated. The quality of life (QoL) and quality of sleep of the patients with BD were found to be impaired, and this may be due to the presence of neuropathic pain syndrome.^2^ A notable study revealed a high prevalence of CS (69.3%) in individuals with BD, highlighting a significant correlation between CS and neuropathic pain, as well as its impact on QoL and disease activity.^3^

Kinesiophobia is a type of fear-avoidance behavior defined as “fear of physical activity and movement” caused by excessive sensitivity and disturbing feelings due to painful situations or repetitive injury.^4^ It usually occurs due to past painful experiences or injuries.^5^ The relationship between CS and kinesiophobia is that the hypersensitive CNS causes physical inactivity as a result of overreacting to stimuli that would not usually cause pain and, as a result, the development of kinesiophobia.^6^ Additionally, studies have shown that CS has a negative effect on exercise capacity. The hypersensitive CNS can cause increased pain during physical activities, reducing the individual’s exercise capacity.^7,8^ This study aims to determine the frequency of CS in patients with BD and evaluate the relationship between CS and exercise capacity, disease activity, and QoL. The secondary objective is to evaluate kinesiophobia and aerobic capacity and analysing the relationship between clinical features, disease activity, and QoL.

## METHODS

We studied consecutive outpatients with BD seen at the rheumatology clinic of the hospital between October 2023 and December 2023. A total of 110 participants, 55 patients with BD and 55 healthy controls (HCs), were included in the study. The inclusion criteria were as follows: patients diagnosed with BD according to International Study Group (ISG) criteria,^9^ participants without previously known systemic chronic cardiac, pulmonary disease, and chronic pain conditions (such as fibromyalgia). Age and gender-matched healthy members of volunteer hospital staff were included as HCs.

### Patient Characteristic and Disease Activity Assessment

The age of the patients, sex, smoking status, educational level, body mass index (BMI), Erythrocyte sedimentation rate (ESR), C reactive protein (CRP), disease duration, age at disease onset, family history of BD, organ involvement, and medications used were recorded. Patients diagnosed with malignancy and any were excluded from the study if they had dysfunctions that limited physical activity, such as severe neurological impairments, immobility, or serious cardiac disorder. QoL was assessed using the validated Behçet’s Disease QoL (BDQoL) questionnaire.^10^

### Behçet’s disease current activity form (BDCAF)

BD severity was assessed using the BDCAF,^11^ a self-reported severity score. The BDCAF was used to measure disease activity over the preceding four weeks. This tool scores organ-specific symptoms, such as mouth and genital ulcers, skin lesions, joint involvement, and gastrointestinal, neurological, or vascular symptoms, on a scale of 0–12. Disease activity was classified as high (≥4) or low (<4). The Turkish version of BDCAF has been validated.^12^

### Behçet’s Syndrome Activity Scale (BSAS)

The BSAS, a 10-item scale assessing disease activity through visual analog scales (VAS, scored 0–10) and categorical measures (scored 0, 5, or 10), was completed by patients. Total scores ranged from 0–100, with higher scores indicating greater disease activity. The Turkish version has been validated as a reliable patient-reported outcome measure.^13^

### Central Sensitisation Inventory (CSI)

Central sensitisation symptoms were evaluated using the 9-item Central Sensitisation Inventory (CSI-9). Each item was scored from 0–4, with a total score range of 0–36.^14^ Scores ≥20 indicated significant central sensitisation.^15^ The Turkish version of CSI-9 has been validated and was used in this study.^16^

### Assessment of functional exercise capacity

Functional exercise capacity was assessed using the 6-minute walk test (6MWT), following European Respiratory Society and American Thoracic Society (ERS/ATS) guidelines. Participants walked a 30-meter corridor for six minutes at their fastest comfortable pace. Resting was allowed but included in the total time. Oxygen saturation, heart rate, and respiratory rate were recorded before and after the test. Perceptions of fatigue, dyspnoea, and quadriceps femoris muscle (QFM) fatigue were evaluated using the Modified Borg Scale (MBS) before, immediately after, and one minute after the test. Walking distances were interpreted using age- and gender-specific norms.^17^

### Tampa Scale of Kinesiophobia – Fatigue (TSK-F)

Kinesiophobia was measured using the Tampa Scale of Kinesiophobia – Fatigue (TSK-F), a 17-item scale adapted for chronic fatigue syndrome by replacing “pain” with “fatigue.” Items were scored on a Likert scale (1–4), with total scores ranging from 17–68.^18^ Scores ≥37 were considered indicative of kinesiophobia.^19^ The Turkish version has been validated and was employed in this study.

### Statistical Analysis

The results obtained are reported as the mean and standard deviation (SD) due to the normal distribution of the sample. The Kolmogorov–Smirnov test was used to assess normality of the distributions. Pearson’s correlations were used for inter-item and item-total correlations because of the normal distribution observed in most cases. Fisher’s exact or Pearson chi-square test was used to compare binary or categorical parameters. Finally, a hierarchical linear regression model was obtained to evaluate the predictors of QoL. Post-hoc power analysis for the sample size of the study was analysed using G*Power 3.1.9.2 software. The kinesiophobia was chosen for post-hoc analysis in the independent sample t-test. The power of the study was found sufficient statistical power (1-β> 95%, α=0.05; d= 1.20; Df= 53). The level of significance was established at p < 0.05. All the statistical analyses were assessed using SPSS software (IBM SPSS Statistics: Version 25, Chicago, IL, USA) and R Studio.

### Compliance with ethical standards

The local ethics committee obtained the study’s ethical approval (Date of approval 07.10.2023, number 23/24). Written informed consent was obtained from the patients before participating in the study. The study was conducted in line with the principles of the Declaration of Helsinki.

## RESULTS

A total of 55 patients (31 males, 24 females) with BD were enrolled in this study. The median (IQR) age of patients was 41(17) years, median disease duration 8.5 (15) years and 65.5% of the patients had low disease activity. The patients with BD were treated with colchicine 40 (72.7%), azathioprine 24 (43.6%), and corticosteroids 8 (14.5%), and four patients were receiving infliximab. The educational background of the participants was primary school 65 (54.9%), high school 22 (17.7%), and higher education 34 (27.4%).

CS was detected in 35 (61.4%) patient with BD compared to 2 (3.6%) in the HC group. When Behçet’s patients were compared with the age-gender-matched HC group, higher CS scores, increased kinesiophobia, and shorter walking distances were observed in patients with BD (**Table 1**). The mean CS score was 21.1±8.7; the TKS score was 42.72±8.9; the 6MWT distance was 461±116 meters in patients with BD, and the CSI was 6.5 (6.18); the TKS score was 25.45±8.01; 6MWT distance 626 ±62 in HCs. (respectively, p values <0.001, <0.001, and 0.011) (**Table 1**). Among the walking test subcomponents, resting dyspnoea, fatigue, muscle fatigue, delta heart rate, QFM fatigue, dyspnoea, and muscle fatigue were significantly higher in the patient group than in the HCs group (**Table 1**).

**Table 1.** Comparison of central sensitisation, kinesiophobia, and 6-Minute Walk Test scores between patients With Behçet’s Disease and healthy controls.

	**Behçet's patients n:55**	**HC n:55**	**p**

Age years, mean±SD	41± 17	33±17	0.129

Gender, female, n (%)	31 (54.4)	29 (52.7)	0.860

Body mass index, mean±SD	25.1±4.15	24.9 ± 3.9	0.794

Smoking status, current n (%)	20 (35.1)	20 (36.4)	0.646

Disease duration, years, median (IQR)	8.5 (15)	-	N.A

Ocular involvement, ever n (%)	20 (36.4)		N.A

Joints involvement, ever n (%)	33 (60)		N.A

Vascular involvement, ever n (%)	16 (29.1)		N.A

Central nervous system involvement, ever n (%)	3 (5.1)		N.A

Medications		-	N.A
Colchicine	40 (72.7%)		
Azathioprine	24 (43.6%)		
Corticosteroids	8 (14.5%)		
Infliximab	4 (0.07%)		

Central sensitisation score, mean (SD)	21.1 (8.7)	6.5 (6.18)	**<0.001**

Central sensitisation, present n %	35 (61.4)	2 (3.6)	**<0.001**

Kinesiophobia score, mean±SD	42.7±8.9	25.4 (8.01)	**<0.001**

6MWT distance, mean±SD	461±116	626±62	**0.011**
Heart rate, beats/min (resting), mean±SD	79.3±11.2	81.2±10.6	0.304
Δ Heart rate, beats/min, mean±SD	23.2 ± 11.3	49.5 (21.8)	**<0.001**
Dyspnoeal (resting), median (IQR)	0(0)	0(0)	**0.019**
Δ Dyspnoea, median (IQR)	0(2)	0(0.5)	**0.002**
Fatigue (resting), median (IQR)	0(0)	0(2)	**0.001**
Δ Fatigue, median (IQR)	1(3)	0(1)	**<0.001**
QFM Fatigue (resting), median (IQR)	0(0.3)	0(0)	**0.005**
Δ QFM Fatigue, median (IQR)	2(4)	0(0)	**<0.001**

6-MWT: 6-minute walk test; HC: healthy control; QFM: Quadriceps femoris muscle; SD: standard deviation; VAS: Visual Analog Scale; Δ: Differences between post-test and pretest values; N.A: not applicable.

Bold values are significant at p<0.05.

While age, age at diagnosis, disease duration, smoking status, educational levels, ESR, CRP, and BMI were similar between the groups, a significantly higher incidence of central CS was observed in females. When comparing the clinical features in patients with BD with and without CS, those with a positive pathergy test and a family history of BD had a higher prevalence of CS (**Table 2**). Additionally, symptoms such as headache, arthralgia, nausea, vomiting, and abdominal pain were more common in patients with CS. No significant differences were found regarding current treatments or comorbidities. When comparing patients with and without CS based on clinical involvement, there were no significant differences in skin-mucosal, joint, vascular, or neurological involvement. However, CS was more frequently observed in those with ocular involvement. Quality of life (QoL) was poorer in patients with CS, and they also had significantly worse BDCAF scores.

Kinesiophobia scores were high, and total walking distance in the 6MWT was significantly lower, and only 6MWT-fatigue score was significantly higher in patients with CS (**Table 2**). When comparing patients based on the presence of kinesiophobia, no significant differences were observed in demographic, clinical, or disease activity characteristics. However, patients with kinesiophobia had lower QoL, higher CSI scores, and reduced walking distance. Notably, within these 6MWT subgroups, only delta fatigue and delta QFM fatigue were found to be elevated (**Table 3**).

**Table 2.** Comparison of demographic and clinical characteristics and disease activity between patients with Behçet’s Disease with and without central sensitisation.

**Variables**	**With CS n:35**	**Without CS n:20**	**p**

Age years, mean±SD	41.7±11.5	41.6±11	0.984

Gender, female, n (%)	22 (66.7)	7 (31.8)	**0.011**

Disease duration, years, median (IQR)	9.5 (14)	5.5 (12)	0.406

Behçet family history, n (%)	13 (39.4)	1 (4.5)	**0.004**

Pathergy test, positive, n (%) n:44	22 (76.8)	5 (31.3)	**0.002**

Oral ulcerations, ever n (%)	32 (97)	18 (81.8)	0.145

Genital ulcerations, ever n (%)	25 (75.8)	15 (68.2)	0.537

Erythema nodosum, ever n (%)	20 (60.6)	10 (45.5)	0.269

Ocular involvement, ever n (%)	16 (48.5)	4 (18.1)	**0.022**

Headache, n (%)	27 (81.8)	11 (50)	**0.012**
Oral aphthous, n (%)	24 (72.4)	12 (54.5)	0.165
Genital ulcer, n (%)	10 (30.3)	2 (9.1)	0.096
Arthralgia, n (%)	27 (81.8)	10 (45.5)	**0.005**
Pustule, n (%)	14 (42.4)	7 (31.8)	0.428
Nausea, vomiting, abdominal pain, n (%)	10 (30.3)	0	**0.004**

BDCAF, median (IQR), (0–12)	3 (3)	2 (7)	**0.018**

PGA, median (IQR), (0–7)	2 (2)	1 (2)	**0.002**

BSAS, median (IQR), (0–100)	24.2 (35.9)	21 (35)	0.869

BD-QoL, mean ±SD, (0–36)	17.8±7.3	9.8±9.1	**0.001**

Kinesiophobia score, mean ±SD	46.3±7.3	37±8.15	**<0.001**

6MWT distance, mean ±SD	423±112.8	519±93.3	**0.002**
Heart rate, beats/min (resting), mean±SD	80.5 (9.9)	77.1 (12.8)	0.277
Δ Heart rate, beats/min, mean±SD	22.6 (11.1)	24.1 (11.6)	0.647
Dyspnoea (resting), median (IQR)	0 (0)	0 (0)	0.405
Δ Dyspnoea, median (IQR)	0 (3)	0 (0.5)	0.129
Fatigue (resting), median (IQR)	0 (3)	0 (1)	0.243
Δ Fatigue, median (IQR)	2 (3.5)	0 (1)	**0.003**
QFM Fatigue (resting), median (IQR)	0 (2)	0 (0)	0.070
Δ QFM Fatigue, median (IQR)	2 (5)	0 (2)	0.060

6MWT: six-minute walk test; BDCAF: Behçet's disease current activity form; BD-QoL: Behçet‘s disease quality of life; bpm: beats per minute; BSAS: Behçet’s Syndrome Activity Scale; QFM: Quadriceps femoris muscle; PGA: Patient Global Assessment; SD: standard deviation; Δ: Differences between post-test and pretest values.

Bold values are significant at p<0.05.

**Table 3.** Comparison of demographic and clinical characteristics and disease activity between patients with Behçet’s Disease with and without kinesiophobia.

**Variables**	**With Kinesiophobia n:41**	**Without Kinesiophobia n:15**	**p**

Age years, mean (SD)	41.7±11.5	41.6±11	0.984

Gender, female, n (%)	24 (58.5)	5 (35.7)	0.140

Disease duration, years, median (IQR)	9.5 (11)	3.5 (18)	0.406

Behçet family history, n (%)	10 (24.4)	4 (28.6)	0.736

Pathergy test, positive, n (%) n:44	21 (63.6)	6 (54.5)	0.724

Oral ulcerations, ever n (%)	32 (97)	18 (81.8)	0.145

Genital ulcerations, ever n (%)	25 (75.8)	15 (68.2)	0.537

Erythema nodosum, ever n (%)	20 (60.6)	10 (45.5)	0.269

Ocular involvement, ever n (%)	15 (36.6)	5 (35.7)	0.953

Headache, n (%)	28 (68.3)	10 (71.4)	1
Oral aphthous, n (%)	28 (68.3)	8 (57.51)	0.552
Genital ulcer, n (%)	9 (22)	3 (21.4)	1
Arthralgia, n (%)	28 (68.3)	9 (64.3)	1
Pustule, n (%)	16(39)	5(35.7)	0.826
Nausea, vomiting, abdominal pain, n (%)	9 (22)	7(7.1)	0.423

BDCAF, median (IQR), (0–12)	3 (3)	2 (2)	0.384

PGA, median (IQR), (0–7)	2 (2)	1.5 (2)	0.397

BSAS, median (IQR), (0–100)	21.5 (26)	30.2 (48)	0.698

BD-QoL, mean ±SD, (0–36)	17±8.2	7.9±7.6	**0.001**

CSI score, mean ±SD	23.12 (7.7)	15.8 (9.6)	**0.006**

6MWT distance, mean ±SD	423 (112.8)	519 (93.3)	**0.011**
Heart rate, beats/min (resting), mean ±SD	80.5±9.9	77.09±12.8	0.703
Δ Heart rate, beats/min, mean ±SD	23.3±10.7	23.1±13.2	0.938
Dyspnoea (resting), median (IQR)	0 (0)	0 (0)	0.297
Δ Dyspnoea, median (IQR)	0 (2)	0 (0)	0.084
Fatigue (resting), median (IQR)	0 (2)	0 (1)	0.547
Δ Fatigue, median (IQR)	2 (3)	0.25 (1)	**0.003**
QFM Fatigue (resting), median (IQR)	0 (1)	0 (0)	0.174
Δ QFM Fatigue, median (IQR)	2 (4.5)	0 (2)	**0.026**

6MWT: six-minute walk test; BDCAF: Behçet‘s disease current activity form; BD-QoL: Behçet‘s disease quality of life; bpm: beat per minute; BSAS: Behçet’s Syndrome Activity Scale; QFM: Quadriceps femoris muscle; PGA: Patient Global Assessment; SD: standard deviation; Δ: Differences between post-test and pretest values.

Bold values are significant at p<0.05.

**Figure 1.**
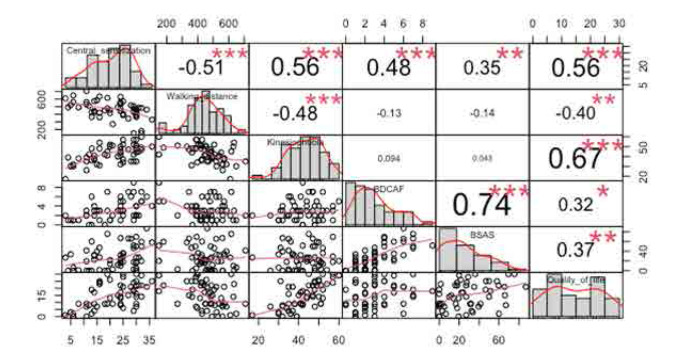
Pearson correlations between central sensitisation and clinical parameters in patients with Behçet’s Disease. BD-QoL: Behçet's disease quality of life; BSAS: Behçet's Syndrome Activity Scale. *p <0.05; **p<0.01; ***p<0.001 Pearson correlations analysis.

Significant correlations were found between CS scores and various clinical parameters, including 6MWT, TKS, BDCAF, and BDQoL scores in patients with BD (Rho = 0.51, Rho = 0.56, Rho = 0.48, and Rho = 0.56; all p values < .001) (**Figure 1**). When the effect of CS, kinesiophobia, and aerobic capacity on QoL was examined in the hierarchical linear regression analysis, a significant relationship was shown with the presence of CS (β:0.298, 95% Confidence interval (CI) 0.033–0.562, p=0.028) and kinesiophobia (β: 0.505, 95% CI 0.245–0.766, p=<0.001) (**Table 4**).

**Table 4.** Hierarchical Linear Regression Analysis (Enter Method): the impact of central sensitisation, kinesiophobia, and aerobic capacity on quality of life in patients with Behçet’s Disease.

**Independent variable**	**Dependent variable**	**β**	**t**	**p**	**95% CI (Lower-Upper)**	**R**	**R Square**	**Significant F Change**

BD-QoL	CSI score	0.586	5.226	**0.028**	0.369–0.823	0.586	0.343	**<0.001**

	CSI score	0.295	2.371	**0.021**	0.045–0.544	0.708	0.501	**<0.001**
	TKS score	0.503	4.048	**<0.001**	0.254–0.752			

	CSI score	0.298	3.899	**0.028**	0.033–0.562	0.708	0.501	0.942
	TKS score	0.505	3.899	**<0.001**	0.245–0.766			
	6MWT	0.001	3.275	0.942	−0.018–0.019			

6MWT: six-minute walk test; BD-QoL: Behçet‘s disease quality of life; CI: confidence interval; Central Sensitisation; TKS: Tampa Kinesiophobia Scale.

Bold values are significant at p<0.05.

Model 1 Central Sensitisation score

Model 2 Central Sensitisation score, Tampa Kinesiophobia Scale Score

Model 3 Central Sensitisation score, Tampa Kinesiophobia Scale Score, and six-minute walk test.

## DISCUSSION

Our study suggests that higher levels of CS in BD are significantly associated with increased kinesiophobia and reduced exercise capacity, both of which negatively impact patients’ QoL. These findings are consistent with research in other inflammatory rheumatic diseases, such as RA, where a positive correlation between disease activity and TKS scores has been reported.^20^ In contrast, systemic lupus erythematosus (SLE) does not exhibit this same relationship, underscoring potential disease-specific differences in the manifestation of kinesiophobia.^21^ Moreover, previous study using computerised techniques showed an increased risk of falling in patients with BD compared to controls, with the risk closely associated with higher disease activity.^22^ These findings further emphasise the interconnected roles of disease activity and CS in determining functional outcomes. However, our study builds on this understanding by specifically exploring how CS impacts kinesiophobia and exercise capacity in BD, shedding light on the vicious cycle of pain, fear of movement, reduced mobility, and diminished QoL. These insights suggest that targeting CS and kinesiophobia through tailored interventions could improve functional capacity and QoL in patients with BD. Combining pharmacological treatments with physical therapy or cognitive-behavioural approaches may help break the cycle of reduced mobility and diminished QoL.

Disease activity in BD is a significant factor influencing patients’ QoL. Our findings are consistent with prior research by Melikoglu et al. and Bodur et al., which emphasise the importance of controlling disease symptoms to improve overall QoL.^23,24^ Furthermore, Ertam et al. demonstrated that chronic conditions like BD are frequently associated with pain, depression, and sleep disturbances, all of which negatively affect QoL.^25^ Together, these studies highlight the complex impact of BD on well-being and underscore the need for comprehensive management strategies to improve QoL in affected patients.

Our study highlights the potential link between increased pain, CS, and a higher risk of falls in patients with BD. CS and kinesiophobia appear to significantly contribute to reduced functional exercise capacity. Heightened pain sensitivity may lead to a reluctance to engage in physical activities, such as walking, as patients fear exacerbating their symptoms. This fear can result in a decrease in walking distance, promoting a sedentary lifestyle that contributes to physical deconditioning and further diminishes walking capacity. The elevated kinesiophobia scores observed in patients with BD are consistent with findings in other inflammatory rheumatic diseases, where fear of movement is a major contributor to functional impairment. In RA, for example, fear-avoidance beliefs have been shown to limit physical activity, as patients avoid movements that they believe may worsen pain or joint symptoms. This avoidance behaviour accelerates functional decline and reduces QoL.^26^ Similar patterns are seen in ankylosing spondylitis, where the fear of exacerbating stiffness and symptoms leads to reduced mobility and worsened function.^27^ In fibromyalgia, fear-avoidance beliefs further perpetuate a cycle of pain and inactivity, as patients avoid physical activity out of fear of triggering more pain, which ultimately results in heightened pain sensitivity and decreased physical capacity.^28^ Our findings align with this broader literature, underscoring the role of fear-avoidance beliefs in limiting physical activity across various rheumatic conditions. Addressing both CS and kinesiophobia through targeted interventions may be critical to breaking this cycle of reduced mobility and improving functional outcomes in patients with BD.

The study’s focus on specific components of the 6MWT, such as pre-test muscle fatigue and post-test general fatigue, adds granularity to our understanding of the factors influencing exercise performance in patients with BD. The association between ocular involvement and increased CS emphasises the intricate relationship between disease manifestations and pain processing, aligning with research highlighting the unique challenges posed by ocular symptoms in BD.^29^ Female gender and family history of BD were associated with a higher likelihood of CS, indicating potential genetic and gender-specific susceptibilities.

Managing CS in BD involves a multimodal approach that addresses pain’s physical and psychological aspects. This may include medications to manage pain and inflammation, physical therapy to improve mobility and function, and psychological interventions such as cognitive-behavioural therapy to address kinesiophobia and promote coping strategies for pain management. Additionally, lifestyle modifications, stress management techniques, and relaxation therapies can also be beneficial in managing CS and improving overall well-being for individuals with BD.

Several limitations should be considered in interpreting our findings. First, the study’s cross-sectional design limits our ability to establish causal relationships between CS, kinesiophobia, and functional outcomes. Longitudinal studies are needed to confirm these associations over time. Second, the relatively small sample size may affect the generalisability of the results, particularly when assessing subgroups such as those with different organ involvement.

In conclusion, the findings of our study regarding CS and kinesiophobia in Behçet’s disease align with broader rheumatic literature, underscoring the need for a holistic approach to patient care. Addressing both the physical symptoms and psychological factors that influence exercise capacity and QoL will be essential in improving the overall management of BD.

## CONFLICT OF INTEREST

The authors declared no potential conflicts of interest with respect to the research, authorship, and/or publication of this article.

## FUNDING

None.

## DATA SHARING STATEMENT

The data supporting this study’s findings are available from the corresponding author (G.A) upon reasonable request.

## AUTHOR CONTRIBUTIONS STATEMENT

All authors contributed to the study’s conception and design. Material preparation, data collection, and analysis were performed by Gülay ALP and Fulden SARI. The first draft of the manuscript was written by Gülay ALP, and all authors commented on previous versions. All authors read and approved the final manuscript.

Conceptualisation: Gülay ALP, Fulden SARI; Methodology: Gülay ALP, Fulden SARI;

Formal analysis and investigation: Gülay ALP, Fulden SARI;

Writing - original draft preparation: Gülay ALP;

Writing - review and editing: Gülay ALP; Fulden SARI; Supervision: Gülay ALP.
